# Treatment effects of psychological interventions on self-harm in individuals with PTSD: A systematic review and meta-analysis protocol

**DOI:** 10.1186/s13643-025-03065-x

**Published:** 2026-01-13

**Authors:** Anthony Tsang, Caroline Clements, Catherine Robinson, Peter J. Taylor

**Affiliations:** 1 Division of Psychology and Mental Health, School of Health Sciences, Faculty of Biology, Medicine and Health, University of Manchester, Manchester, UK; 2 Division of Nursing, Midwifery and Social Work, School of Health Sciences, Faculty of Biology, Medicine and Health, University of Manchester, Manchester, UK

## Abstract

**Background:**

There is robust evidence reflecting that individuals with PTSD are significantly more at risk of engaging in self-harm and suicidal behaviours. Trauma-focused interventions for PTSD, however, predominantly focus on PTSD symptomology-related outcomes. Therefore, there is a dearth of knowledge regarding the treatment effects of psychological interventions in individuals with PTSD examining self-harm-related outcomes. This evidence synthesis will identify studies that investigate interventional effects on self-harm, non-suicidal self-injury (NSSI), and suicide attempts.

**Methods:**

A comprehensive bibliographic search will be conducted to identify eligible randomised controlled trials (RCTs) and non-RCT evaluation studies indexed in Ovid MEDLINE, Embase (Ovid), PTSDPubs (ProQuest), APA PsycInfo (Ovid), PubMed (NOT MEDLINE[SB]), Web of Science Core Collection (CPCI-SSH), CENTRAL, WHO ICTRP, and ClinicalTrials.gov from inception to present day. A range of supplementary search techniques will also be employed to reduce the file drawer effect. Two independent reviewers will screen records at a title/abstract and full-text level, with 50% extracted data being cross-checked by an independent reviewer. Eligible studies will be assessed for risk-of-bias (RoB) using Cochrane’s RoB 2.0 for RCTs and ROBINS-I for non-RCTs. A combination of random- and fixed-effects meta-analytic models will be performed separately for RCTs and non-RCTs, and for post-intervention and follow-up periods for self-harm, NSSI, and suicide attempts separately and in aggregate, using *d*-family effect sizes (including Hedges’ *g*) and risk ratio/odds ratio for continuous and binary outcome data, respectively, with associated 95% confidence intervals. Sensitivity analyses will be performed to examine if methodological decisions impact on summary effects. Potential sources of heterogeneity will be examined as moderators (e.g. adults versus non-adults, intervention delivery, and complex PTSD versus PTSD) using mixed-effect meta-regression models. The evaluation of certainty of evidence of all main outcomes will be conducted using the GRADE approach. The review will be reported in accordance with PRISMA-S and PRISMA 2020 guidelines.

**Discussion:**

This systematic review and meta-analysis of RCTs and non-RCTs will provide a comprehensive synthesis of treatment effects of psychological interventions on self-harm related outcomes among individuals with PTSD. The results may increase a better understanding of which interventions are best suited to targeting self-harm outcomes in this population. The completed review will be published in a peer-reviewed journal.

**Systematic review registration:**

PROSPERO CRD42024598594

**Supplementary Information:**

The online version contains supplementary material available at 10.1186/s13643-025-03065-x.

## Introduction

Post-traumatic stress disorder (PTSD) is a debilitating mental health condition primarily characterised by (i) intrusive re-experiencing (e.g. nightmares, flashbacks, and memories) of the traumatic experience; (ii) heightened arousal and reactivity (i.e. hyperarousal); (iii) avoidance of associated stimuli; and (iv) negative changes in associated cognitions and mood; where exposure to a traumatic event involves actual or threatened death, serious injury, or sexual violence [1] or is appraised as extremely threatening or horrific [2]. PTSD is associated with high rates of self-harm, with prevalence estimates often exceeding 50% [3, 4]. In fact, children with PTSD have eight times higher odds of engaging in self-harm and ten times higher odds of suicide attempts compared to their peers without PTSD [5]. While antecedents such as childhood sexual abuse increase this risk [6, 7], the development and maintenance of self-harm are driven by the mediating role of core PTSD symptoms [8–13] in conjunction with disturbances in self-organisation, specific to complex-PTSD (C-PTSD) [14]. Critically, C-PTSD symptomology is known to be particularly robust risk factors for self-harm, as individuals are more likely to engage in such behaviours to regulate emotional distress [15–17].

Various psychological interventions are available for the treatment of C/ PTSD. For both prevention and treatment, trauma-focused (TF) interventions have been recommended for adults, which include cognitive processing therapy [18], cognitive behavioural therapy (CBT) [19], eye movement and desensitisation and reprocessing (EMDR) [20], narrative exposure therapy, and more recently prolonged exposure [21]. For children, group TF-CBT interventions and individual TF-CBT are recommended for prevention and treatment, respectively [22]. Notably, while clinical guidelines acknowledge the additional complexities of C-PTSD, there is currently no standalone intervention protocol exclusive to this condition [22]. Whether a clinical difference in self-harm outcomes exists between PTSD and C-PTSD following treatment has yet to be empirically established. To date, evidence syntheses and randomised controlled trials (RCTs) evaluating the effect of interventions for PTSD primarily focus on outcomes related to changes in PTSD symptomology. These include trauma-related appraisals [23], symptom exacerbation during treatment [24], the impact of single versus multiple traumatic events [25], and PTSD symptom severity [26–31]. These findings emphasise the predominant focus on symptom changes in PTSD research, leaving other important outcomes such as self-harm unexamined.

Despite the plethora of evidence syntheses examining the effect of interventions in PTSD-based populations, no existing review has yet to measure self-harm-related outcomes. Indeed, one of the most common exclusions used in RCTs of psychological treatments for PTSD is suicidal ideation with intent [32]. Although emerging RCTs and other study designs are beginning to address this major limitation, research often focuses on interventions that are less commonly recommended for PTSD and self-harm [33–36]. Additionally, a recent review on self-harm outcomes focused solely on psychosocial interventions, which did not target populations with PTSD [37]. Therefore, it remains unclear how suitable recommended treatments are for reducing self-harm frequency and severity in individuals with PTSD. Evidence specifically examining self-harm-related outcomes in PTSD samples is diverse in terms of interventions and study design. Examples include an RCT investigating the effects of dialectical behaviour therapy [38], a pilot study examining EMDR [39], and a secondary data analysis of cognitive processing therapy [40]. Given the diversity of interventional studies and the high prevalence of self-harm in individuals with PTSD, a comprehensive systematic review of the evidence landscape is a priority to identify and synthesise such studies and help inform and update pre-existing clinical guidelines and improve patient care. Therefore, the overall aim of this review is to investigate the treatment effects of psychological interventions in reducing self-harm behaviours among individuals with PTSD. The specific objectives will be to as follows: (i) investigate the treatment effects of psychological interventions on different typologies of self-harm, including overall self-harm, and non-suicidal self-injury (NSSI), and suicide attempts; (ii) ascertain the certainty of evidence (i.e. confidence in effect estimates) associated with the reduction of overall self-harm, NSSI, and suicide attempts; and (iii) examine potential moderators on outcomes using meta-regression.

## Method

### Protocol registration and reporting

The present study review adheres to the reporting guidelines of Preferred Reporting Items for Systematic Reviews and Meta-Analyses Protocols (PRISMA-P) [41] (see checklist in Additional File 1). The completed review will be reported in accordance with the PRISMA literature search extension (PRISMA-S) checklist [42] and PRISMA 2020 guidelines [43]. This protocol is registered in the International Prospective Register of Systematic Reviews (PROSPERO) with the registration number CRD42024598594 [44].

### Eligibility criteria

Adults (18 years or older) and non-adults (17 years or younger) with PTSD or C-PTSD diagnosis (including comorbidities such as major depression) according to all versions of DSM and ICD will be included. Full or probable diagnosis of PTSD or C-PTSD based on medical records, clinical interviews, or other validated screening tools which are adequately described for replication will also be considered for inclusion. Self-reported diagnosis will be excluded, alongside individuals with substance-induced PTSD, intellectual disabilities, or traumatic brain injury only.

Psychological intervention is defined as a treatment programme that aims to produce a change via psychological processes or mechanisms without the use of medicinal products or devices [45–47]. All psychological interventions that are designed or used for those with PTSD and/or C-PTSD will be included, regardless of frequency or duration of sessions. This includes, but is not limited to, dialectical behavioural therapy, eye movement desensitisation and reprocessing, and trauma-focused cognitive behavioural therapy. All modes of intervention delivery, including a variety of combinations, will be included, such as clinician-driven, self-help based, and online-based.

The primary outcome of interest in this review is self-harm and is defined as an intentional act of self-poisoning or injury, irrespective of the motives or apparent purpose of the act [48]. Suicide attempts, NSSI, and other typologies of self-harm will be included. Other forms of suicidal behaviour such as aborted or interrupted suicide attempt(s) will also be included. Self-harm operationalised as binary (e.g. presence of self-harm) and continuous (e.g. severity or frequency of behaviour) variables using any type of measures (e.g. Suicide Attempt Self-Injury Interview, [49]; Deliberate Self-Harm Inventory [50]) will be included. Death by suicide and reckless and self-destructive behaviours (RSDBs; e.g. reckless driving and substance abuse) will be excluded as they are conceptually distinct from self-harm. If relevant aspects of self-harm are explicitly reported separately in the measure of RSDBs, these will be included. Unintentional injury and outcomes that combine suicidal behaviour and ideation will be excluded.

All peer-reviewed and non-peer-reviewed studies will be eligible. Controlled and uncontrolled trials or treatment evaluation studies using either a between-group and within-group approach will be included. Mixed-method studies will be included if there is a quantitative component reporting relevant statistical data. Conference abstracts and protocols will only be included if there is an accompanying full-text report available. Relevant systematic reviews will also be screened for additional studies. Qualitative study designs, case studies, editorials, commentaries, and letters will all be excluded.

Studies with or without a control group will be included if individuals with PTSD or C-PTSD constitute 50% or more of the total sample. For studies that include treatment focused on a single intervention (e.g. TF-CBT) and treatment arms that include the same single intervention in combination with other interventions (e.g. CBT plus mindfulness), the arm focused on the single intervention alone will be included. Additionally, combined intervention studies (e.g. psychological plus pharmacological) will be ineligible unless data for the psychological component are reported independently, allowing for the isolation of its effects. Finally, all languages will be included and online translation tools (e.g. DeepL) will be used for non-English studies. To ensure translation accuracy, key high-level summary characteristics (e.g. key outcomes and sample characteristics) will be back-translated into English to verify the original translation. If the translation of studies is considered unreliable or inaccurate following this process, these will be excluded.

### Search strategy

Prior to commencing this evidence synthesis, a search was conducted on PROPSERO and Google Scholar (limited by 2014–2024) to identify any pre-existing reviews on this topic area to avoid duplication. The development of the search syntax was facilitated through a series of activities. Iterative informal scoping searches were conducted in PTSDPubs (ProQuest) and Google Scholar to identify relevant studies used to facilitate the development of the search syntax for the review. Single word and multiple-word frequency analysis was conducted on WriteWords [51] using the titles and abstracts of relevant studies to identify relevant free-text terms. In addition, unique record identifiers of these studies were used in PubReMiner [52] to perform a frequency analysis of subject headings. Upon identification of relevant subject headings, Emtree and entry terms were consulted to identify further potential search terms. Following preliminary testing on Ovid MEDLINE, a search using two search concepts was developed and will be systematically translated across the other databases and trial registries with the assistance of MEDLINE Transpose [53] and Polyglot Search Translator [54]. One search concept pertains to PTSD and the other pertains to self-harm. A combination of subject headings (or their equivalent, e.g. Emtree terms on Embase) and free-text terms has been used. A separate search concept pertaining to publication types (e.g. editorials, commentaries) has been incorporated at the end of the search syntax for removal, using the Boolean operator NOT in the final line. No language or date limits will be applied to the searches. Limits will only be used to deduplicate records from other databases and no filters will be used. The overall search strategy and search syntax were peer-reviewed by a librarian and the last author (PJT). The Peer Review of Electronic Search Strategies (PRESS) checklist was used as a final step of validation [55]. An example of the search syntax is available in Additional File 2.

A systematic bibliographic search will be performed on the following databases and trial registries: PTSDPubs (ProQuest), Ovid MEDLINE, Embase (Ovid), APA PsycINFO (Ovid), PubMed (NOT MEDLINE[sb]), Web of Science Core Collection (CPCI-SSH), CENTRAL, WHO ICTRP, and ClinicalTrials.gov. Although there is significant overlap between Ovid MEDLINE and PubMed [56], the decision to include both databases is two-fold: (i) to circumvent the limitations of search functionalities of PubMed (e.g. absence of frequency operator) and (ii) studies of interest published in journals indexed in the PubMed Central subset of PubMed. All searches will be performed from the inception of each database/trial registry to the present day.

In an attempt to reduce the file-drawer effect [57] and circumvent the limitations of database searching, purposive supplementary searches will be undertaken following specific steps of the CLUSTER searching technique [58]—Citations, Lead authors, Unpublished materials, Scholar searches, Theories, Early examples, and Related projects. This approach is recommended for retrieving studies that may have been missed in database searches and for identifying similar and/or related studies in systematic reviews [59]. The first step of CLUSTER will be to identify a “key pearl” such as trial identification numbers. Forward and backward citation searches (steps 2 and 5) will be performed on all eligible studies using citationchaser [60]. Searches of institutional repositories and personal webpages/publication webpages of key authors of eligible studies (step 4) will be conducted. Also, searches of project name/identifier (step 6) of potentially relevant protocols (where applicable) on Google and Google Scholar for other potential related projects (step 12) will be considered. Finally, unpublished literature will be sought via key author contact (step 7), for example, to determine accompanying full texts of conference abstracts, and ProQuest Dissertations and Theses Global will be searched with the sole intention to find unpublished literature.

### Screening procedure and management

All search results from bibliographic databases will be downloaded and imported into EndNote (version × 9) for deduplication. The process of deduplication of records will involve a two-fold sequential process: (i) the in-built automatic detection of duplicates will be used in EndNote and (ii) manual identification following a modified, systematic approach of Bramer et al. [61] that will involve changing field settings and filters in the settings in EndNote. After deduplication, the remaining records will then be imported into Covidence [62] for screening.

A two-step screening process will be adopted. For search results deriving from the primary searches (i.e. bibliographic databases and trial registries), two independent reviewers will first screen titles and abstracts, followed by full-texts. In contrast, one reviewer (AT) will initially screen results originating from supplementary searches (i.e. steps from the CLUSTER approach). The decision as to which records will be opened and further examined will be based on the reviewer’s judgement. Full-text reports of records will be retrieved via EndNote’s “Find Full Text” function (missing full-text reports will be sought for manually) and subsequently uploaded to Covidence. Reports will then be screened by two independent reviewers. All discrepancies in screening decisions will be resolved via discussion among the two reviewers. If a resolution cannot be reached, a third independent reviewer will arbitrate. A PRISMA 2020 flow diagram will be completed to depict the results of the searches and screening process, and show reasons for exclusion at full-text level.

### Data extraction

Data for each included study will be extracted by one reviewer (AT), while a second independent reviewer will cross-check a random 50% subsample as a pragmatic measure. Any disagreements will initially be discussed between the primary extractor and the cross-checker. A third independent reviewer will arbitrate if disagreements cannot be resolved. Data extraction will cover the following high-level study characteristics to facilitate the contextualisation of the included studies: (i) study (e.g. author, year of study, and design); participants (e.g. sample size, type of trauma, age, gender, method of diagnosis, and severity of diagnosis); outcomes (e.g. measure(s) of self-harm, suicidal behaviour, self-harm methods); intervention (e.g. name of intervention, mode of delivery, and summary of content); and statistics (e.g. treatment-related effect sizes, means, and standard deviations). Data from full-text reports will be prioritised over corresponding conference abstracts and posters. The authors will be contacted if there are any missing data or ambiguity between different sources of data. The contacted authors will have 4 weeks to reply before a follow-up email will be sent. If authors do not respond, missing data will be clearly stated, and the potential impact on the overall findings will be discussed.

### Assessment of risk of bias and certainty of evidence

Two reviewers will independently perform risk of bias and Grading of Recommendations, Assessment, Development, and Evaluation (GRADE) [63] assessments. For RCTs, the risk of bias of each relevant outcome will be evaluated using the Cochrane Risk-of-Bias (RoB) 2.0 [64], which examines five domains: “bias due to randomisation process”, “deviations from intended intervention”, “missing data outcome”, “measurement of the outcome”, and “selection of the reported result”. For non-RCTs, the risk-of-bias in non-randomised studies—of interventions (ROBINS-I) tool [65] (or ROBINS-I 2.0 for follow-up studies [66]) will be used to assess RoB per outcome. ROBINS-I evaluates seven domains across three intervention phases: pre-intervention (“bias due to confounding” and “bias in selection of participants”); intervention (“bias in classification of interventions”); and post-intervention (“bias due to deviations from intended interventions”, “bias due to missing data”, “bias in measurement of outcomes”, and “bias in selection of reported result”). When using RoB 2.0, the overall RoB judgments will be classified as low RoB, some concerns, or high RoB. For ROBINS-I, the overall RoB judgments will be categorized as low RoB, moderate RoB, serious RoB, critical RoB, or no information. RoB assessments will be conducted using a pre-specified excel tool for RoB or template for ROBINS-I. A visualisation tool for RoB assessments will be used to illustrate all results [67].

The GRADE approach will be used to evaluate the certainty of evidence among main outcomes (i.e. self-harm, NSSI, and suicide attempts) through consideration of five domains (“risk of bias”, “inconsistency”, “indirectness”, “imprecision”, and “publication bias”) [68]. There are four levels of certainty (high, moderate, low, and very low), and outcomes deriving from RCTs can be downgraded if there are serious limitations within the five domains. GRADE will be conducted separately for RCTs and non-RCTs for the same outcomes. For example, if RoB is deemed high for evidence from RCTs, this would lower the certainty from high to moderate. For evidence from non-RCTs, certainty begins at low but can be upgraded through a further three domains (“large effects”, “dose–response”, and “plausible confounding”), which can also be applied to RCTs. An adapted GRADE guideline will be followed for outcomes without a single estimate of effect [69]. A summary of findings (SoF) table will be developed that provides key information related to the magnitude of effects for outcomes, the certainty of evidence, and key information to contextualise findings. GRADE and SoF will be conducted using GRADE’s software GRADEpro guideline development tool [70]. Any discrepancies in risk-of-bias or GRADE assessments will be resolved via discussion or arbitrated with a third independent reviewer until resolution.

### Methods of evidence synthesis

All effect size calculations, transformation of effects, and analyses will be carried out for outcomes reported at both post-intervention and follow-up using the statistical software R (version 4.3.1 or newer) [71] and the integrated development environment, RStudio (version 2024.09.01 + 394 or newer) [72]. The R packages meta [73], metafor [74], dmetar [75], esc [76], fishmethods [77] and MOTE [78] are anticipated to be used for effect transformation, calculation, and pooling, and meta-regression. The main effect size metric will be Cohen’s d-family effects (including Hedges’ g to bias-correct for small samples) for continuous outcome data, whereas risk ratios (RRs) or odds ratios (ORs) will be the main effect for binary data. It is anticipated that two main approaches will be used for effect size computation. First, uncalculated effect size data will be prioritised if reported statistics allow for calculation of effects (e.g. descriptives such as means, sample sizes, and standard deviation) for both between- and within-group studies. Second, pre-calculated effects will be used in the absence of raw data using other computational methods if *d*-family effects or RRs/ORs are not reported. For example, t values will be used to estimate d-based effects. To pool studies using pre-calculated effects, reported 95% confidence intervals (CIs) will be used to calculate a standard error (SE) using the recommended formula by Cochrane: SE = (upper CI limit—lower CI limit)/3.92 [79]. The interpretation of d-family effect sizes will follow the frequently used convention of small (0.2), medium (0.5), and large (0.8) [80]. The interpretation of RR and OR will be the same, albeit the use of language will differ based on the specific effect (e.g. odds-related language for ORs and risk/probability-related language for RRs). The following convention will be used: RR/OR of 1 will mean the exposure (i.e. intervention) does not affect the outcome, RR/OR greater than 1 will mean the risk/odds of the outcome is increased by the exposure, and RR/OR less than 1 will mean the risk/odds of the outcome is decreased by the exposure [81].

Based on relevant studies identified from scoping searches, self-harm is expected to be measured as a binary (e.g. presence of self-harm) or continuous outcome (e.g. severity or frequency of behaviour), and these will be synthesised separately. Additional steps will be undertaken to ensure consistency and maximise comparability across studies: (i) the sign of effects will be consistently coded in the same direction to facilitate the interpretation of results. For example, a negative value such as *d* = –0.5 would indicate a favourable result for the intervention in reducing self-harm, (ii) adult and non-adult studies will be pooled together. However, if the largest proportion is marginal (e.g. 51% versus 49%), such studies will be omitted during sensitivity analyses, (iii) where studies do not provide data for total samples, summary statistics for multiple subgroups (e.g. males and females) will be combined into a single group before synthesis. For binary outcomes, sample sizes and the number of individuals with events will be summed across groups. For continuous outcomes, means, standard deviations, and sample sizes will be combined using the recommended Cochrane formula (see Additional File 3) [79], (iv) composite outcomes will be prioritised over subscales to ensure independence of effects, (v) Cohen’s d_av will be calculated for within-group study designs to account for the effect of different experimental designs, which uses the average, instead of pooled standard deviation as the standardiser [82]. This will allow direct comparison with classical Cohen’s d for between-group studies [82, 83], (vi) for binary outcomes, a continuity correction of 0.5 will be added when the total number of events in a specific column is zero across all included studies [84], (vii) categorical data with more than two levels (e.g. low, moderate, and high levels of severity of self-harm) will be dichotomised (presence/absence of self-harm) to allow equivalent computation of effects, and viii) post-intervention will be defined as the first time-point after the end of the intervention window, and follow-up will be time-points after post-intervention, and will be defined by three time-frames: short (4 months or less), medium (between 5 and 8 months), and long (beyond 8 months). Each follow-up time-frame will have its own synthesis, if data is available.

A combination of random- and fixed-effects meta-analytic models will be applied to conduct three primary analyses: (i) aggregated self-harm (including NSSI and suicide attempts), (ii) NSSI alone, and (iii) suicide attempts alone. Each analysis will be separated into RCTs and non-RCTs, as well as post-intervention and follow-up. For continuous data, random-effects models will employ a generic inverse-variance method using the restricted maximum likelihood estimator [85], which is recommended to estimate between-study heterogeneity for such data [86]. Knapp–Hartung adjustments [87] will be applied to control for the uncertainty in the estimate of between-study variance and to avoid false positives, especially when there are a limited number of studies [88, 89].

For binary outcome data, the fixed-effect exact Mantel–Haenszel method [90] will be used due to its superior statistical properties when there are few events and the anticipated small-sized studies [91]. Another advantage of the exact Mantel–Haenszel method is that it eliminates the need for continuity corrections, except in cases where an entire cell column contains zero events [91]. For rare events (< 1%), such as suicide attempts, the Peto inverse-variance fixed-effects method may be considered if the number of events is similar across study groups and the treatment effect is not excessively large [92]. However, fixed-effect models may be omitted in the event of significant heterogeneity (based on the *I*^*2*^ statistic) or absence of raw data. Instead, an inverse-variance random-effects model using the Paule–Mandel estimator [93] will also be performed as recommended [86].

Heterogeneity analyses will be performed for all main analyses. Heterogeneity will be identified using *Tau*^*2*^ to estimate between-study variance in random-effect models [91], whereas the Cochran’s *Q*-statistic (chi-squared test), which assesses whether differences in results are compatible with chance alone [91] will be used for fixed-effect models. The *I*^*2*^ statistic will be used to quantify the magnitude of inconsistency for all models, describing the percentage of total variability in effect estimates. The thresholds of interpretation of *I*^*2*^ will be based on the following: low (25%), moderate (50%), and high (75%) [94]. Ninety-five percent prediction intervals will be calculated to provide the dispersion in effects of future studies [95].

Alternative methods of synthesis may be employed based on data availability when a meta-analysis is not feasible [96]. These methods include summarising the distribution of observed effects (e.g. using descriptive statistics such as median, interquartile range, and range) or combining p-values in the case of limited reported statistical data, or vote-counting based on the direction of effect. These methods will be incorporated with a modified narrative synthesis approach [97], where a sequential, three-step approach will be undertaken: (i) preliminary synthesis of studies (e.g. tabulation and transforming data into a common metric); (ii) examining relationships within and between studies (e.g. comparing characteristics of studies such as intervention type, population, and measures of self-harm); and (iii) assessing the robustness of the synthesis (e.g. using risk of bias results to contextualise and explain results).

### Examination of moderators and ancillary analyses

Mixed-effects meta-regression will be considered where at least ten studies are available with sufficient covariate variability to investigate independent moderators on summary effects [91]. This model is suitable due to its flexibility that accounts for within-study and between-study variance [98]. The categorical variables will include diagnosis of complex PTSD versus PTSD, intervention delivery (e.g. clinician-driven, self-based, and online-based), intervention type (if sufficient variation exists), adults versus non-adults, and the classification method of PTSD diagnosis (e.g. ICD or DSM) as the disorder is defined differently [99]. Multiple meta-regression models using the forced entry approach will be adopted to examine the influence of a moderating variable while controlling for confounders. The maximum likelihood estimator will be employed as this permits comparison of meta-regression models to identify the best fit [74]. Multicollinearity will be examined using the variance inflation factor (VIF). VIF values greater than 10 will indicate the presence of multicollinearity, and in such instances, the variables will be reported and a decision of which variable to remove will be discussed among team members. Knapp-Hartung adjustments will be used to obtain more robust estimates by reducing the risk of false significant effects [87]. Finally, permutation tests will be run to assess the robustness of a model in resampled data. Such tests will be reported before the results of a meta-regression model as recommended [100]. However, permutation tests will only be run if the number of studies is greater than four in order to reach statistical significance [101].

Ancillary analyses will consist of the following: (i) outlier and leave-one-out analyses will be conducted to examine the robustness of summary effects, (ii) sensitivity analyses will be performed to remove any identified outliers or influential studies (e.g. non-peer-reviewed and mixed population studies), and (iii) publication bias analyses will be conducted for outcomes with a recommended minimum of ten studies [102], and will be examined through visual inspection of funnel plots and quantified using the Egger’s test [103]. In the presence of significant publication bias (*p* < 0.05), the trim-and-fill method will be used to estimate the potential influence of hypothetically missing studies [104].

## Discussion

This systematic review and meta-analysis will provide treatment effects on psychological interventions for people with PTSD who self-harm, using trial and evaluation study designs. It will investigate whether PTSD-targeted therapies reduce overall self-harm, NSSI, and suicide attempts or if self-harm specific approaches are required instead. By evaluating treatment effects, the findings may elucidate which interventions yield the greatest reductions in self-harm related outcomes. Additionally, the review will highlight research gaps, such as with limited or inconsistent evidence regarding intervention types and delivery methods, and assess confidence in the findings by identifying potential methodological limitations in existing studies. The synthesis of the current evidence landscape will identify key therapeutic targets for future interventional studies. These insights can guide clinicians in optimising treatment approaches to improve patient care by reducing distress and self-harm. Moreover, it supports policymakers in prioritising interventions where evidence is strongest, potentially helping to reduce costs by focusing resources on the most effective treatments instead of providing a broad range of potentially less effective options.

A key methodological strength of this review protocol is the comprehensive study eligibility criteria, as it does not impose the common limitations on geography, date, or language often seen in other reviews. Although this review is including both RCTs and observational studies, it is anticipated that a potential limitation is that the majority of eligible studies will be observational in nature.

The completed review will outline and justify any amendments or deviations to this protocol. All statistical analyses and data used, including the R code, will be provided in an R markdown document to ensure open science and transparency. Given the significant public health burden of PTSD and its strong association with self-harm, this review will provide a rigorous synthesis of the available evidence. The results will attempt to determine whether self-harm in PTSD populations is amendable to current trauma-focused interventions, thereby informing the clinical needs of this population.

## Supplementary Information

Additional file 1.

Additional file 2.

Additional file 3.

## Data Availability

The datasets during and/or analysed during the current study are available from the corresponding author on reasonable request.
