# Correction: Treatment effects of psychological interventions on self‑harm in individuals with PTSD: a systematic review and meta‑analysis protocol

**DOI:** 10.1186/s13643-026-03211-z

**Published:** 2026-05-25

**Authors:** Anthony Tsang, Caroline Clements, Catherine Robinson, Peter J. Taylor

**Affiliations:** 1 Division of Psychology and Mental Health, School of Health Sciences, Faculty of Biology, Medicine and Health, University of Manchester, Manchester, UK; 2 Division of Nursing, Midwifery and Social Work, School of Health Sciences, Faculty of Biology, Medicine and Health, University of Manchester, Manchester, UK

**Correction: Syst Rev 15, 54 (2026)**

**https://doi.org/10.1186/s13643-025-03065-x**

In the sentence beginning 'Heterogeneity analyses will be performed for all main analyses…' in this article, the symbol for '*I*^*2*^' was displayed incorrectly as I2 when it should have been *I*^*2*^. Furthermore, the same goes for the capturing of “Tau2” it should be “*Tau*^*2*^”.

The original article [1] has been corrected.
