# Supplementary material for: Treatment effects of psychological interventions on self-harm in individuals with PTSD: A systematic review and meta-analysis protocol
Source: Syst Rev. 2026 Jan 13;15:54. doi: 10.1186/s13643-025-03065-x (PMC12888150; doi:10.1186/s13643-025-03065-x)
Supplement: Supplementary file 2 — Additional file 2. [file 13643_2025_3065_MOESM2_ESM.docx]

**Additional File 2**

**Search syntax for Ovid MEDLINE (R)**

**PTSD search concept:**

1. “Sexual Trauma”/ or “Military Sexual Trauma”/ or “Combat Disorders”/ or “Trauma and Stressor Related Disorders”/ or “Stress Disorders, Traumatic”/ or “Psychological Trauma”/ or “Historical Trauma”/ or “Stress Disorders, Traumatic, Acute”/ or "Stress Disorders, Post-Traumatic"/ or “compassion fatigue”/ or (Post Traumatic Stress$ or Posttraumatic Stress$ or PTSD).ti,ab,kf /freq=2 or (Acute Post-Traumatic Stress Disorder or Acute Posttraumatic Stress Disorder or Chronic Post-Traumatic Stress Disorder or Chronic Posttraumatic Stress Disorder or Delayed Onset Post-Traumatic Stress Disorder or Delayed Onset Posttraumatic Stress Disorder or Moral Injur$ or Posttraumatic Neuros#s or Post-Traumatic Neuros#s or traumatic neuros#s or posttraumatic psychosis or post traumatic psychosis or posttraumatic syndrome or post traumatic syndrome or traumatic stress or war neuros#s or sexual trauma$ or sexual abuse trauma$ or sexual assault trauma$ or acute stress or psychological$ trauma$ or Emotional trauma or trauma symptom$).ti,ab,kf or ((trauma adj3 associated adj3 stimuli) or (trauma adj3 related adj3 stimuli)).ti,ab. or (traumatic adj2 (event$ or incident$ or situation$)).ti,ab,kw,kf. or (combat stress or shell shock or combat neuros#s or combat fatigue or combat disorder$).ti,ab,kf or ((Re experienc$ adj3 symptom$) or (Reexperienc$ adj3 symptom$) or (Trauma$3 adj5 reexperiencing) or (Trauma$3 adj5 re experiencing) or (Trauma$3 adj3 nightmare$) or (Trauma$3 adj3 flashbacks) or (Trauma$3 adj3 emotional respons$) or (Trauma$3 adj3 hyperalert$) or (Trauma$3 adj3 hyper alertness) or (symptom$ adj3 hyper alertness) or (symptom$ adj3 hyperalertness) or (Trauma$3 adj4 intrus$4) or (Hyperarousal adj3 symptom$) or (Hyper arousal adj3 symptom$) or (Hyper arousal adj3 trauma$3) or (Hyperarousal adj3 trauma$3) or (Increase$ adj2 arousal) or (Numb$ adj2 emotion$) or (Avoidance adj3 trauma$3) or (Avoidance adj2 symptom$)).ti,ab,kf.

**Self-harm and suicidal behaviour concept:**

1. ((Poisoning/ or "wounds and injuries"/ or weapons/ or firearms/) and ("Self-Injurious Behavior"/ or "Self Mutilation"/ or "Suicide, Attempted"/)) or *Self-Injurious Behavior/ or *Self Mutilation/ or *Suicide, Attempted/ or (NSSH or NSSI or Self-Injur$ or selfinjur$ or Self-harm$ or Selfharm$ or Self-destructive Behavio?r$ or nonsuicidal behavio?r$ or non suicidal behavio?r$ or self-inflicted violence or DSH or self-abuse or self mutilat$ or selfmutilat$ or automutilation or self-directed violence or self-inflicted injur$ or self-wounding or self-inflicted mutilation or self-inflicted harm or selfinflicted injur$ or selfinflicted wound$ or non suicidal injur$ or self inflicted wound$ or self-hit$4 or Cutting).ti,ab,kf. or ((Self adj2 burn$) or (Self adj2 poison$) or (Head adj3 bang$3)).ti,ab,kf. or (((blunt object or sharp object) adj5 (self-harm or self-injury)) or ((deliberate or direct) adj destruction adj2 body tissue)).ti,ab. or suicidal behavio?r.ti,ab,kf. /freq=2 or (Non fatal adj3 behavio?r$).ti,ab. or (parasuicid$ or (Suicid$2 adj1 attempt$)).ti,ab,kf.

**Publication type**

1. (Comment or Case reports or News or Address or Newspaper article or Personal narrative or Editorial or Letter).pt

**Final line**

1. (1 and 2) not 3
