# Supplementary material for: Treatment effects of psychological interventions on self-harm in individuals with PTSD: A systematic review and meta-analysis protocol
Source: Syst Rev. 2026 Jan 13;15:54. doi: 10.1186/s13643-025-03065-x (PMC12888150; doi:10.1186/s13643-025-03065-x)
Supplement: Supplementary file 3 — Additional file 3. [file 13643_2025_3065_MOESM3_ESM.docx]

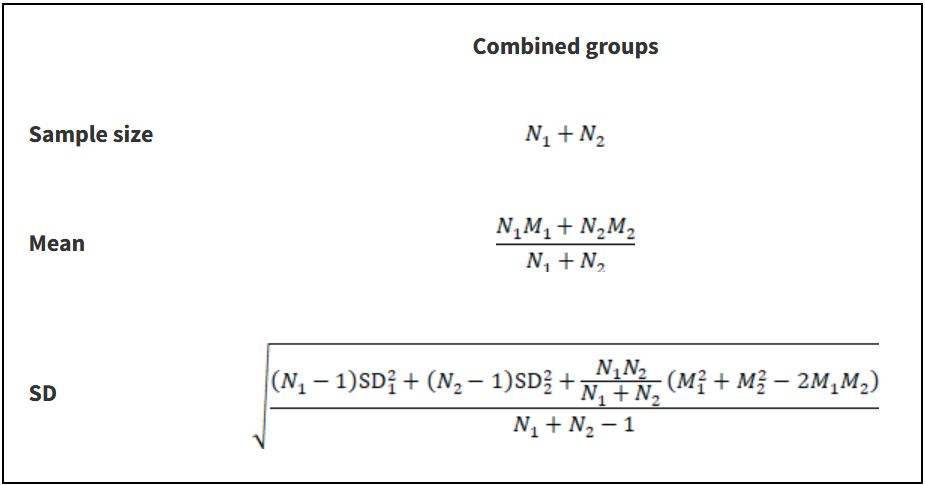
**Additional File 3**

This illustration is derived from the following: Higgins JPT, Li T, Deeks JJ. Chapter 6: Choosing effect measures and computing estimates of effect [last updated August 2023]. In: Higgins J.P.T, Thomas J, Chandler J, Cumpston M, Li T, Page MJ, et al., editors. Cochrane Handbook for Systematic Reviews of Interventions version 65. 2024.
